# A multivariate method to determine the dimensionality of neural representation from population activity

**DOI:** 10.1016/j.neuroimage.2013.02.062

**Published:** 2013-08-01

**Authors:** Jörn Diedrichsen, Tobias Wiestler, Naveed Ejaz

**Affiliations:** Institute of Cognitive Neuroscience, University College London, UK

**Keywords:** Multivoxel pattern analysis, Representational models, Primary motor cortex

## Abstract

How do populations of neurons represent a variable of interest? The notion of feature spaces is a useful concept to approach this question: According to this model, the activation patterns across a neuronal population are composed of different pattern components. The strength of each of these components varies with one latent feature, which together are the dimensions along which the population represents the variable. Here we propose a new method to determine the number of feature dimensions that best describes the activation patterns. The method is based on Gaussian linear classifiers that use only the first *d* most important pattern dimensions. Using cross-validation, we can identify the classifier that best matches the dimensionality of the neuronal representation. We test this method on two datasets of motor cortical activation patterns measured with functional magnetic resonance imaging (fMRI), during (i) simultaneous presses of all fingers of a hand at different force levels and (ii) presses of different individual fingers at a single force level. As expected, the new method shows that the representation of force is low-dimensional; the neural activation for different force levels is scaled versions of each other. In comparison, individual finger presses are represented in a full, four-dimensional feature space. The approach can be used to determine an important characteristic of neuronal population codes without knowing the form of the underlying features. It therefore provides a novel tool in the building of quantitative models of neuronal population activity as measured with fMRI or other approaches.

## Highlights

• Neural population activity represents external variables using multiple feature dimensions.
• We present a general method to determine the dimensionality of this feature space.
• Primary motor cortex represents force through a low-dimensional feature space.
• Individual finger movements are represented using a four-dimensional feature space.

## Introduction

One of the key questions in neuroscience is how populations of neurons represent the world surrounding us. Representation here is defined in a broad sense: it simply implies that the activity of a certain population of neuronal units (neurons, cortical columns, or voxels) contains information about a variable that characterizes an object or the state of the world, for example the identity of a face or the velocity of an arm movement. While relatively commonplace in neurophysiology, representational analysis approaches are increasingly applied also to functional magnetic resonance imaging (fMRI) data (for reviews, see Haynes and Rees, 2006; Tong and Pratte, 2012). The novel aspect of this class of analyses is that it asks questions regarding neural representation, rather than neural activation. If individual voxels have different tuning functions over the possible values of the variable, then the cortical area can represent the variable faithfully without showing any overall differences in mean activation level between conditions (Wiestler et al., 2011). Therefore, multivariate analysis can detect representations that may not be visible to more traditional mass-univariate analysis approaches (i.e. Friston et al., 1994).

Decoding models, especially classification, have proven to be very useful as methods to establish whether the activity of an ensemble of neuronal units contains information regarding (i.e. represents) a certain variable (Pereira et al., 2009). In classification approaches, one attempts to predict the value of the represented variable – often in terms of discrete experimental conditions – based only on the activity pattern of a certain cortical region. The classifier is trained on a subset of the data and is subsequently tested on the remaining set (cross-validation). Above-chance classification accuracy therefore implies that different conditions lead to reliably different activity patterns within the region. Following our definition, this implies that the region represents the variable in question.

**Fig. 1:**
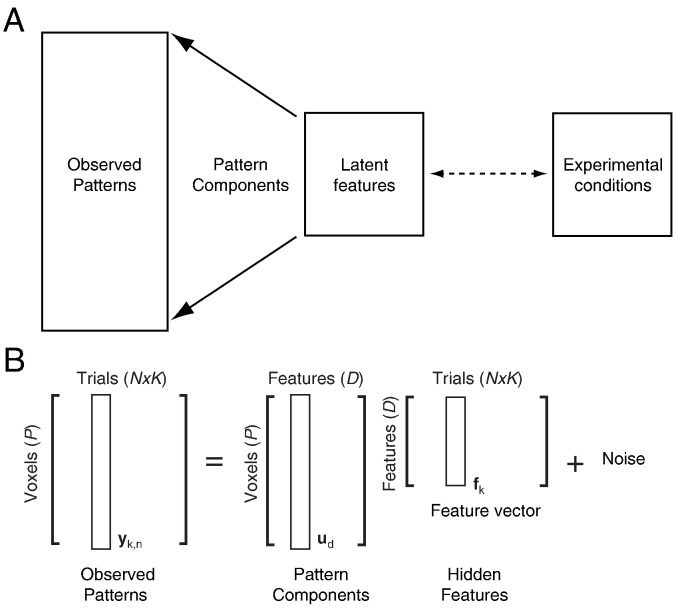
Latent feature spaces. (A) In the theoretical framework, the observed patterns of neural activity are explained by a set of latent features, each of which is linearly combined with an associated pattern component. The mapping between the experimental conditions (stimuli, movements, tasks, etc.) and the features (dashed line) can be non-linear. (B) Mathematically, each observed pattern (**y***_k,n_*) is a linear combination of different pattern components (**u***_d_*), each weighted by the corresponding dimension in the latent feature vector (**f***_k_*).

However, from the neuroscientific point of view, we would not only like to determine *whether* activity in a region represents a certain experimental variable, but also *how* the region does so. In approaching this question, it is useful to consider the general framework depicted in Fig. 1A (Naselaris et al., 2011). The core idea is that the connection between the measured neural activation patterns and the experimental conditions is mediated by a set of latent (hidden or unobserved) features that the region represents. Each feature is associated with a specific pattern component, and the observed activity patterns are the sum of different pattern components (Diedrichsen et al., 2011), weighted by the value of the corresponding feature (Fig. 1B). The mapping from experimental conditions to features is unknown and can be any arbitrary non-linear function. In this framework, the question of how a region encodes certain experimental conditions is equivalent to trying to characterize the space spanned by the latent features.

**Fig. 2:**
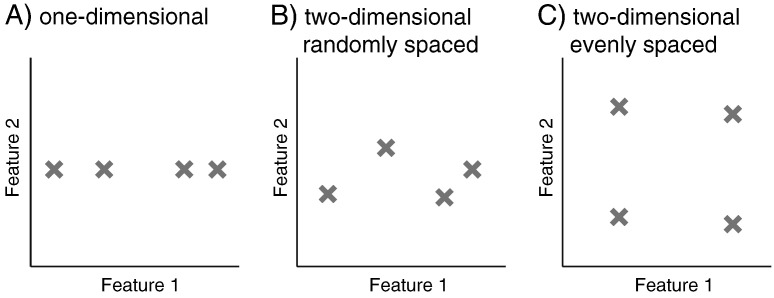
Conditions can be differently arranged in feature space. Shown are the values of four conditions on two feature dimensions. Each feature dimension is associated with a pattern component, which determines the mean activity pattern for each condition. (A) When all four conditions only differ on a single feature dimension, only one dimension in pattern space will be necessary to distinguish them. (B) If the conditions are randomly arranged in feature space, the second pattern component will contribute less to the distinguishability of the associated patterns than the first feature dimension. (C) If the conditions are evenly spaced, then each associated dimensions in pattern space will contribute equal amounts to the distinguishability of the conditions.

One important characteristic of a feature space is its dimensionality: the number of feature dimensions, and hence the number of corresponding pattern components, that underlie the observed activation patterns (*D*, in Fig. 1B). Take as an example a neural population that represents the direction and amplitude of reaching movements (Eisenberg et al., 2010; Schwartz et al., 1988) via a set of units that show cosine tuning with the angle of movement direction. Thus, each unit would show maximal activity for its preferred direction, the lowest activity the opposite direction, with intermediate activity for the remaining directions. A population of such units (each with its own preferred direction and modulation depth) would represent different planar movements in term of two latent features (in this case the length of the x- and y-components of the movement). Thus, if we recorded the neural activity of this region during planar reaching movements in 4 different directions, then these movements should lead to activation patterns that are equally spaced in a two-dimensional feature space (Fig. 2C).

However, due to the anisotropy in the biomechanics of the arm (Bhushan and Shadmehr, 1999), some of these movement directions will require more force and effort than others. Thus, classification could also yield above-chance accuracy if the neural activity in a region only varied with the exerted effort, rather than with movement direction per se. In this case, the region would “represent” the four conditions by virtue of one single variable (i.e. effort, or any nonlinear function thereof). Consequently, the four experimental conditions would vary only along one pattern component (Fig. 2A). Thus, by measuring the dimensionality of the representation, we can test whether a region has a full representation of movement direction (regardless of the shape of this representation), or whether a region simply distinguishes between the different directions by the virtue of a single unknown variable.

Finally, it is important to consider that differences in dimensionality are quantitative, rather than qualitative. For example, if the conditions are randomly spaced in feature space (Fig. 2B), then one dimension will (by chance) separate the conditions better than the other. This representation would therefore occupy an intermediate position between a one-dimensional (Fig. 2A) and a two-dimensional representation with evenly spaced conditions (Fig. 2C). Therefore, we can characterize representations not only by the *number* of dimensions, but also by how the information is distributed across these dimensions.

In this paper, we will first formalize the idea of pattern components, latent features, and feature spaces. We will then introduce a method, based on the Gaussian linear classifier, to determine the underlying dimensionality of the representation, and validate the method using Monte-Carlo simulations. We will then turn to two empirical examples, one in which the underlying feature space is relatively low-dimensional, and a second one which has a full-dimensional representation.

## Methods

### Generative model and notation

We consider here experiments that measure the neural response in *K* experimental conditions. Each condition is measured *N* times, to a total of *N × K* trials. The *n*th measurement of population activity for condition *k* is denoted by the *P × 1* vector **y**_*k*,*n*_, which contains all *P* features (voxels, neurons, electrodes, time points) that describe the measurement on that trial.

The generative model (Fig. 1B) assumes that each measured activity pattern is a linear combination of different pattern components, each of which represents a certain feature of the underlying representations. To formalize this relationship, each experimental condition is associated with one feature vector *f*_*k*_, whose elements form the dimensions of the feature space. The length of this vector (*D*) therefore defines the dimensionality of the feature space. The observed activity patterns **y**_*k*,*n*_ are linear combinations of the pattern components **u**_*d*_ (*P × 1* vectors) each weighted by the *d*th element of the latent feature vector for condition *k* (*f*_*k*_^(*d*)^) plus random noise:

(1) $$y_{k,n}=\sum_{d=1}^{D}u_{d}f_{k}^{\left(d\right)}+\varepsilon_{k,n}\text{.}$$

Thus, each pattern component expresses the change in the pattern of neural activity for a given change in the value of the corresponding element of the feature vector. We consider pattern components to be random vectors (Diedrichsen et al., 2011), drawn independently from the same distribution. This is simply for convenience; the introduction of a covariance structure across pattern components can be equally translated into a change in the distribution of conditions in the feature space. Under these assumptions, the expected (or mean) value of the neural response for each experimental condition is

(2) $$\mu_{k}=\sum_{d=1}^{D}u_{d}f_{k}^{\left(d\right)}\text{.}$$

### Reduced-dimensional linear classifier

Here we propose a method to determine the dimensionality of the feature space. The core idea of the method is to use a series of Gaussian linear classifiers (for an introduction, see Duda et al., 2001), each of which uses only the *d* most important dimensions of the training data to classify the test data in a cross-validated approach. Classifiers that use fewer dimensions than present in the representation will perform poorly, as they neglect important information. Classifiers that use more dimensions than necessary will over-fit the data and equally perform poorly. Optimal classification performance will ideally be achieved only by the classifier that uses the correct number of dimensions. In this section, we will first derive the standard (or full-dimensional) Gaussian linear classifier and then show how to reduce its dimensionality.

The generative model for the Gaussian linear classifier assumes that the activity patterns over *P* voxels for each condition – in the context of classification typically referred to as classes – are distributed as multivariate normal random variables with a common *P × P* covariance matrix ∑_*W*_, and class-specific *P × 1* mean pattern *μ*_*k*_.

The Gaussian linear classifier assigns a new pattern **y** (a measured *P × 1* vector) to the class *k*, for which the (log-) probability is highest that **y** was drawn from the corresponding class distribution. This log-probability can simply be derived from the fact that the patterns for each class have multivariate normal distribution:

(3) $$\begin{array}{l} \log\;p\left(y|k\right)=-\frac{1}{2}{\left(y-\mu_{k}\right)}^{T}\;\sum_{W}^{-1}(y-\mu_{k})-c\\ =\mu_{k}^{T}\;\sum_{W}^{-1}y\;-\frac{1}{2}\mu_{k}^{T}\;\sum_{W}^{-1}\mu_{k}\;-\underset{\mathrm{independent}\;\mathrm{of}\;k}{\underbrace{\frac{1}{2}y^{T}\;\sum_{W}^{-1}y\;-c}}\text{,} \end{array}$$

where c is a collection of constant terms. Note that the bracketed term does not depend on the class *k* and can therefore be dropped for the classification.

Assuming we have measured *N* trials for each condition *k*, we train the classifier on a set of *N ∗ K* data points by obtaining estimates of the expected class means (${\hat{\mu }}_{k}$), and the covariance matrix (${\hat{\Sigma }}_{W}$).

(4) $$\begin{array}{l} {\hat{\mu }}_{k}=\frac{1}{N}\sum_{n=1}^{N}y_{k,n}\\ {\hat{\sum }}_{w}\;=\frac{1}{\left(N-1\right)K}\sum_{k=1}^{K}\sum_{n=1}^{N}\left(y_{k,n}-{\hat{\mu }}_{k}\right)\;{\left(y_{k,n}-{\hat{\mu }}_{k}\right)}^{T}\text{.} \end{array}$$

Because the estimate of the within-class covariance, ${\hat{\Sigma }}_{w}$, is usually rank-deficient (because *P* ≥ (*N* − 1)*K*), we regularize it by adding a small constant to the diagonal, here 1% of the mean of the diagonal elements. While this method is relatively ad-hoc, it reliably produces good cross-validated accuracies on real neural data (Diedrichsen et al., 2012; Wiestler et al., 2011) and often outperforms more principled forms of regularization (Ledoit and Wolf, 2003).

By plugging in these estimates into Eq. (3) we can obtain a discriminant function for each class (*g_k_*, see below) and assign the new pattern **y** to the class with the highest *g_k_*.

(5) $$\begin{array}{l} g_{k}\left(y\right)=h_{k}y-\frac{1}{2}h_{k}{\hat{\mu }}_{k}\\ h_{k}\;=\;{\hat{\mu }}_{k}^{T}{\hat{\sum }}_{W}^{-1} \end{array}$$

where **h**_*k*_ is a (column-) discrimination vector that can be pre-calculated to increase computational speed. As we can see from this derivation, a Gaussian generative model results in the well-known Fisher linear discriminant analysis (LDA), in which the classification function is linear in the data vector **y**.

The new method is based on a series of linear classifiers, each using a reduced number of pattern dimensions. These pattern dimensions can be most easily found using an alternative representation of the data. We therefore first transform the data and the condition means into a pre-whitened space, by multiplying them with the inverse square root of the covariance matrix: y* = *Σ*_*W*_^− 1/2^**y**, and *μ*_*k*_^⁎^ = *Σ*_*W*_^− 1/2^*μ*_*k*_. In this new space, the within-class variance is spherical, var(y*) = **I** and the log-likelihood (Eq. (3)) equals (up to a constant) the squared Euclidian distance between a pattern and the group means.

(6) $$\begin{array}{c} \log\;p\left(y^{*}|k\right)=-\frac{1}{2}{\left(y^{*}-\mu_{k}^{*}\right)}^{T}\;(y^{*}-\mu_{k}^{*})-c\\ =\mu_{k}^{*T}y^{*}-\frac{1}{2}\mu_{k}^{*T}\mu_{k}^{*}-\frac{1}{2}y^{*T}y^{*}-c\text{.} \end{array}$$

To capture the spacing of the group means in this space, we now calculate the between-class covariance matrix (*P × P*):

(7) $$\begin{array}{c} \sum_{B}^{*}\;=\frac{1}{K}\sum_{k=1}^{K}\left(\mu_{k}^{*}-\mu^{*}\right)\;{\left(\mu_{k}^{*}-\mu^{*}\right)}^{T}\\ =\frac{1}{K}\sum_{k=1}^{K}\Sigma_{W}^{-1/2}\left(\mu_{k}-\mu \right)\;{\left(\mu_{k}-\mu \right)}^{T}\;\Sigma_{W}^{-1/2}\text{.} \end{array}$$

where *μ* is the mean activation pattern (*P* × 1) across all classes in the original space, and μ* is the mean pattern in pre-whitened space. Given this result, we can now determine how well any arbitrary direction in the pre-whitened space distinguishes between the classes. For this we project the whitened class means onto any vector **w**, and calculate the between-class variability for these projected values:

(8) $$S\left(w\right)=\frac{1}{K}\sum_{k=1}^{K}w^{T}\left(\mu_{k}^{*}-\mu^{*}\right)\;{\left(\mu_{k}^{*}-\mu^{*}\right)}^{T}w\;=\;w^{T}\Sigma_{B}^{*}w\text{.}$$

Thus, larger values of *S(***w***)* indicate a better separation between the classes or conditions. To find the vector that separates the classes best, we therefore have to find the **w** that maximizes *S*(**w**). This is the classical eigenvalue problem, in which the eigenvectors of ∑_*B*_^⁎^ are the *d* orthogonal directions (**w**) that provide optimal separation of the conditions.

Consider a case in which the underlying feature space is truly one-dimensional. In this scenario, we note that the separation along any other dimension orthogonal to the pattern component associated with the feature will be zero, i.e. we should find only one non-zero eigenvalue. If, however, the patterns differ along more than one dimension, then for each additional separating dimension, an additional eigenvalue will be non-zero. Maximally, we can find *K*-1 non-zero eigenvalues for *K* classes: we call this a *full representation*.

On empirically measured data sets, all *K*-1 eigenvalues will be non-zero. This is because the measurements are noisy, and due to these random fluctuations the patterns will differ, at least slightly, along all possible dimensions. Since there is no closed-form solution for the size and distribution of eigenvalues under certain dimensionalities, we propose to use cross-validation to estimate the true number of dimensions.

In this scheme, we repeatedly divide the data set into a test set and training set, typically by taking one imaging run as a test set, and using the remainder of the data from that participant as a training set. The parameters of the classifier are then estimated on the training set (Eq. (4)), and used to classify the measured patterns (**y**, *P* × 1 vectors) of the test set. To reduce the classifier to *d* dimensions, we project the data and the estimated class means onto the eigenvectors associated with the *d* highest eigenvalues. Let the columns of matrix **W**^(*d*)^ be the *d* = *1 … K-1* eigenvectors of the estimated between-class covariance matrix in pre-whitened space (${\hat{\sum }}_{B}^{*}$), then the new *d*-dimensional classification functions are:

(9) $$\begin{array}{c} g_{k}^{\left(d\right)}\left(y\right)={\left(W^{\left(d\right)T}{\hat{\mu }}_{k}^{*}\right)}^{T}\;W^{\left(d\right)T}y^{*}-\frac{1}{2}{\left(W^{\left(d\right)T}{\hat{\mu }}_{k}^{*}\right)}^{T}\;\left(W^{\left(d\right)T}{\hat{\mu }}_{k}^{*}\right)\\ =h_{k}^{\left(d\right)}y-\frac{1}{2}h_{k}^{\left(d\right)}{\hat{\mu }}_{k}\\ h_{k}^{\left(d\right)}={\hat{\mu }}_{k}^{T}{\hat{\Sigma }}_{W}^{-1/2}W^{\left(d\right)}W^{\left(d\right)T}{\hat{\Sigma }}_{W}^{-1/2}\text{.} \end{array}$$

We then apply each *d*-dimensional classifier to the independent test set. This method is then repeated, each time selecting a different run to serve as test set. Given sufficient data, the classifier that matches the dimensionality of the underlying feature space should have the best classification accuracy. Classifiers with fewer dimensions should miss essential features of the data; classifiers with more dimensions will capture all information, but the additional dimension will cause more noise in the discriminative functions (Eq. (9)), ultimately leading to more misclassifications (over-fitting).

### Generation of simulated data

To generate artificial data, we first defined a feature space to have dimensionality *D*. We then assigned each of the *K* experimental conditions a “true” value in this feature space, i.e. for each condition we generated a vector **f**_*k*_ of length *D*. Two approaches were used: For randomly distributed feature vectors (Fig. 2B), we drew the feature vector for each condition from a multivariate standard normal Gaussian (mean zero, identity covariance matrix). Secondly, we also generated the feature vectors such that each feature dimension provided equal separation between conditions (Fig. 2C). To achieve this, we first generated a feature space with as many dimensions as experimental conditions by setting the *k*th element of each feature vector **f***_k_* to 1, while the other elements were set to zero. We then concatenated these column vectors into a matrix **F** and subtracted the mean of each row, resulting in a matrix with values of *1 − 1/K* on the diagonal, and − *1/K* elsewhere. We then projected the feature vectors onto *D* eigenvectors of **F** (each having an identical and non-zero eigenvalue). This method ensures in general (for any *D* ≤ *K*) that all *D* feature dimensions are independent and provide the same amount of separation between *K* conditions.

After generating a latent feature vector for each experimental condition, we then generated for each feature dimensions a pattern component vector, **u**_*d*_. These *P* × 1 vectors were independently drawn from a multivariate normal distribution with 0 mean and variance *Iσ*_*a*_^2^. This variance *σ*_*a*_^2^ determines how strongly the features are represented (relative to the noise) in the fMRI signal. For all simulations, we assumed that the patterns are independent across the P voxels (or neuronal units); however all results also hold when a spatial covariance is introduced (Diedrichsen et al., 2011).

**Fig. 3:**
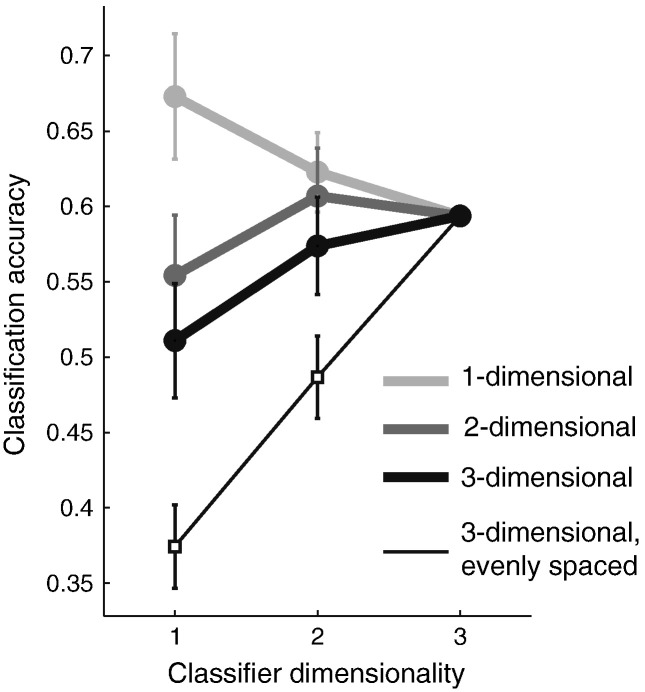
Proportion of accurate classifications for the 1, 2, and 3-dimensional classifiers (x-axis) on simulated datasets generated from a 1- (light gray), 2- (middle gray), or 3-dimensional (thick black line) feature space with random arrangement of the conditions. A simulation with features that were spaced maximally in 3-dimensional feature space is shown as a thin black line. Simulation runs are selected to have the same accuracy on the full classifier (0.58). Error bars indicate the 95% confidence interval when drawing and averaging over 40 independent simulation runs.

**Fig. 4:**
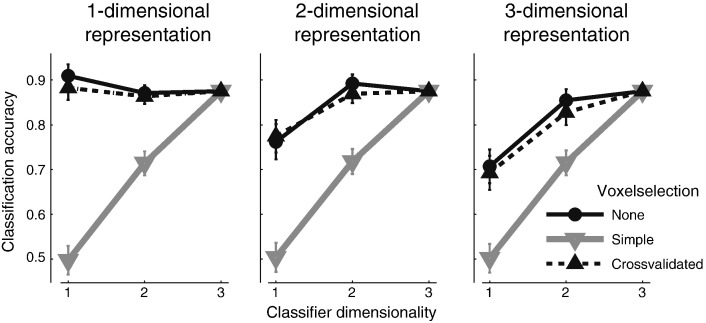
Influence of voxel-selection on dimensionality analysis. Classification accuracy for the 1- to 3-dimensional classifier (x-axis) for simulated data, assuming a 1-, 2- or 3-dimensional representation. Simple voxel-selection (choosing the 15% most informative voxels from an area based on an overall F-test) seriously biases the accuracy curve. Voxel-selection nested within the cross-validation (dashed line) does not have the same adverse effect.

For each dimensionality and spacing of features, we generated 10,000 simulation runs. We systematically varied the variance parameter *σ*_*a*_^2^ between 0 and a value that gave us a classification accuracy of 100% for the full-dimensional classifier. To be able to compare the classification accuracy curves between simulated data set of different dimensionality, we selected for Figs. 3 and 4 only simulation runs that had a fixed value for the full-dimensional classifier. For comparison with the empirical data, we matched group of voxels with the simulation runs that had the same accuracy for the full-dimensional classifier (see Random subspace method section).

### General MRI methods

All participants were scanned on a 3 T Siemens Trio system, which was equipped with a 32-channel head coil. Functional data was acquired using a 2D echo-planar imaging sequence with 32 interleaved slices and an isotropic resolution of 2.3 mm (TR = 2.72 s). Field-maps were obtained after the first functional run to correct for inhomogeneities in the primary magnetic field (Hutton et al., 2002). For each of the participants, we also acquired a single T1-weighted anatomical scan (3D MPRAGE sequence, 1 mm isotropic, 240 × 256 × 176 mm FOV).

To measure the isometric forces generated by each digit of the hand, we used a custom-built response box with five keys, each of which was equipped with a force transducer (FS-series, Honeywell). The keys measured force up to 20 N with a repeatability of < 0.05 N. The force transducer signals were low-pass filtered at the magnetic room wall to avoid the ingress of RF-noise into the MRI environment, and subsequently amplified and digitized outside the magnet room. The response keyboard was firmly placed on the lap of the participant, and tilted towards them at a ~ 45° angle. Visual stimulus was provided to the participants via a back-projection screen through a mirror mounted on top of the head-coil. The Ethics committee of University College London approved all experimental procedures.

### Representations of force levels (Experiment 1)

Five participants volunteered for this experiment. They were healthy and right handed (3 females, 2 males, mean age = 23 years, SD = 6.44). During the fMRI scan participants were visually instructed to produce different force levels through isometric key presses of all fingers of the right hand. The forces over all fingers were summed together. As a target for this summed force we used the four different force levels of 5, 10, 20, and 40 N, corresponding to 1, 2, 4 and 8 N per finger on average. While the distribution of forces across fingers did not matter, participants were instructed to distribute the forces over all five fingers in a comfortable, natural manner. Analysis of the behavioral data shows that participants mostly scaled a single force pattern. A square (~ 1 by 1°) in the center of the screen changed the brightness proportional to the sum of all finger forces that participants applied to the keyboard. A single trial (10.8 s, 4TRs) consisted of four presses of the same force level. Each press was cued with the presentation of 24 circular pie segments (diameter 15° visual angle) around the central square. Alternating wedges were assigned brightness proportional to the target force, while the others were black. The segments alternated between black and the brightness with a frequency of 10 Hz to provide optimal stimulation to primary visual cortex. Participants were instructed to match the brightness of the center square to the brightness of the surrounding segments. After 1.6 s, the visual stimulus was switched off, and participants stopped pressing and relaxed their fingers. After 1.1 s, the next press began. We acquired a total of 8 imaging runs, each consisting of 16 trials (four per force level) in pseudo-random order. We also randomly intersperse five rest phases of 16.2 s length, causing each run to be 4.59 min (102 TRs) long.

### Representation of individual finger presses (Experiment 2)

The data set has been published and the main methods described in detail (Experiment 1, Diedrichsen et al., 2012). Six right-handed healthy young participants (2 females, 4 males, mean age = 25.9 years, SD = 5.1) were scanned while making isometric key presses on the force measurement board. Each trial lasted 3 TRs (8.16 s) and started with a presentation of an instruction stimulus that indicated which finger of which hand had to be pressed. The stimulus persisted on the screen for 1.36 s before being removed. After this short instructional cue, the visual display was identical for the different trial types. The central fixation-cross then turned into the white letter “P”, which was the go signal to press the selected finger. When the force on the instructed finger exceeded 2.3 N, a response was recognized and the central letter “P” turned blue. A total of five go-signals per trial were presented 1.36 s apart. Each of the ten fingers was probed three times during each imaging run, resulting in 30 trials per run. Additionally we inserted five rest phases of 5–6 TR lengths, during which the participants only fixated a central fixation cross, resulting in 126 TRs per run. We collected eight imaging runs per participant. For the analysis presented here, we used the data from both the left and right motor cortex, but only for trials in which a contralateral finger was pressed.

### Basic data analysis

The functional data was analyzed using SPM 8 (Friston et al., 1999) and custom-written Matlab code (Mathworks, Natick, MA). In order to allow for time so that the MRI response builds up to steady state, the first three volumes of each imaging run were discarded. The different slices were temporally re-aligned to account for the interleaved slice acquisition. Images were corrected for field inhomogeneities and head motion using a six-parameter rigid-body alignment (Hutton et al., 2002). We then constructed a first-level linear model with a design matrix with a separate regressor for each condition, which took the value of one during a certain trial type and zero otherwise. Each run was modeled by a set of independent regressors. These boxcar regressors were then convolved with the standard hemodynamic response function. The design matrix and data were both high-pass filtered with a cutoff period of 128 s. The design matrix also included a separate intercept for each imaging run. The resultant regression coefficients (beta-weights) therefore measured the relative change in BOLD signal for a specific condition within an imaging run, and were taken as input data (**y**) for the classification analysis. The linear model was estimated on unsmoothed data.

To visualize the data, we reconstructed the cortical surface of each participant using the Freesurfer software package (Dale et al., 1999). These surfaces were then inflated and aligned to a standard spherical surface to optimally superimpose the cortical folding pattern across participants (Fischl et al., 1999). To define regions-of-interest (ROIs) we used probabilistic cyto-architectonic maps, which were equally aligned to the standard cortical surface. We defined motor-cortical ROIs (Fischl et al., 2008) by combining the two maps for Brodmann area 4 (BA4, rostral and caudal). The surface-based ROI was then projected onto the individual gray–white matter and pial surface. For each participant, we then selected all voxels that touched either of these surface patches (or lay between the surface patches) to be contained in the ROI. The resulting BA4 ROI contained on average 1290 voxels per hemisphere (SD = 209 voxels).

### Random subspace method

To improve the reliability of the classification accuracies for the classifiers of different dimensionality, we utilized a random subspace approach. From the voxels of the BA4 ROI, we drew repeatedly sets of 80 voxels (2000 drawings). These subsets were then submitted to the classification analysis (see Reduced-dimensional linear classifier section), and the classification accuracies over all 2000 drawings were averaged. To generate the comparisons curves for known distributions, we selected for each drawing of the real data all simulation runs with the same accuracy for the full-dimensional classifier. The predicted accuracy curve for each drawing was the average performance of the lower-dimension classifier for these simulation runs, and the overall predicted accuracy was the average of these curves across the 2000 subspace drawings.

### Projection of patterns into the feature space

Finally, to visualize the arrangement of different conditions in estimated feature space (${\hat{f}}_{k}$), we can project the measured mean patterns onto the first *d* eigenvectors of *Σ*_*B*_^⁎^. For this we can arrange the eigenvectors as columns into the matrix **W**^(*d*)^. The projected values then are

(10) $${\hat{f}}_{k}=W^{\left(d\right)T}\sum_{W}^{-1/2}{\hat{\mu }}_{k}\text{.}$$

In this projected space, the Euclidian distance between group means corresponds to the Mahalanobis distance in the original space, and the between-condition variability along each of the feature dimensions corresponds to the eigenvalues. To compare feature spaces across multiple participants, we aligned the different projections for scaling, rotation and translation using Procrustes analysis (Schönemann, 1966).

## Results

### Simulation result

To establish the validity and sensitivity of the method, we simulated neural data for an experiment with *K* = 4 conditions. We created data sets for which the underlying feature space had either *D* = 1, 2, or 3 dimensions. The feature vectors for each condition were generated by drawing from a *D*-dimensional normal distribution. We also randomly generated *D* random pattern components with variability *σ*_*a*_^2^ (see Generation of simulated data section). The simulated activity patterns resulted from weighting each random pattern component by the value of the corresponding feature and adding independent noise for each trial (Eq. (1)). Over the 10,000 simulation runs for each dimensionality, we systematically changed the variability of the pattern components (*σ*_*a*_^2^), such that we could select examples with different dimensionality that had comparable classification accuracies for the full classifier.

The generated data was then subjected to the proposed cross-validated classification analysis (see Reduced-dimensional linear classifier section), reserving 1/8 of the data as a test set, and repeating the process 8 times. Each time, we used two reduced-dimensional classifiers (1- and 2-dimensional), and one full classifier (3-dimensional). Each classifier used the training set (subset of data from 7 runs) to determine the *d* (1, 2, or 3) most important dimensions, and then predicted the condition for each pattern in the test set (subset of data from a single run), using only these dimensions. For the chosen classifiers, we expected that the cross-validated accuracy for the different simulations would be highest if the dimensionality of the classifier matched the dimensionality of the underlying data.

As can be seen in Fig. 3 this was indeed the case. For data generated from a one-dimensional feature space (light gray line), the one-dimensional classifier showed better accuracy than the 2- or 3-dimensional classifier. The latter two performed worse, as they over-fit the data: the additional dimensions contained random noise, and therefore induced classification errors. Averaged over all the simulation runs with a one-dimensional representation, the one-dimensional classifier had the highest accuracy in 68% of the cases, followed by the two-dimensional (18%) and the three-dimensional classifier (14%).

Similarly, the 2- and 3-dimensional data sets were – on average – best classified using a 2- and 3-dimensional classifier, respectively. However, the accuracy curves (middle and dark gray line) were much more similar to each other with only 41% of these simulation runs having the best accuracy for the correct classifier. The main reason for this overlap is that the 4 conditions are not evenly spaced in the 3-dimensional feature space. Rather, due to their random placement, the first two dimensions contained most of the between-condition variance. In other words, four randomly placed points in 3d space can be relatively well described by a plane. The eigenvalues of the true between-category variance–covariance matrix (∑_*B*_^⁎^, Eq. (7)) indicate how much variability each dimension, in descending order of importance, explains. The normalized eigenvalues were [1 0 0] for the one-, [1 0.29 0] for the 2- and [1 0.39 0.09] for the 3-dimensional simulation. This means that for 4 randomly placed conditions in a 3-dimensional feature space, the third dimension comprises only 9% of the between-class variance contained in the first dimension, and only 0.09 / (1 + 0.39 + 0.09) = 6% of the total variance. Therefore, adding a third dimension to the classifier does not substantially improve classification accuracy. To determine the classification curve if all dimensions are equally important, we added a simulation in which the four conditions were evenly distributed in 3-dimensional feature space (eigenvalues [1 1 1], see Generation of simulated data section). For this arrangement, the advantage of the 3-dimensional classifier (Fig. 3, thin black line) over the 1- or 2-dimensional classifier became much clearer.

### Comparing full accuracy curves

The results in the preceding section indicate that we can, in principle, identify the most likely feature space dimensionality of a given population activity by determining the dimensionality of the best classifier. While this method appears to work well for distinguishing between one-dimensional and higher-dimensional representations, the simulations also show that the 2- and 3-dimensional representations cannot be distinguished reliably. However, Fig. 3 indicates that that there is substantial information in the full accuracy curves that is not reflected in the maximum. For example, the accuracy curves for the 2- and 3-dimensional representations do not only differ in the likely dimensionality of the maximum, but also in the expected accuracy of the one-dimensional classifier for a given accuracy of the full classifier. Furthermore, the randomly and evenly spaced representations differ only in the accuracy of the 1-, and 2-dimensional classifier. Thus, we prefer to display and evaluate the full accuracy curves, rather than just their maximum. For this approach it is necessary to generate simulated accuracy curves under a specific model of how much each feature dimension contributes to the discrimination between conditions (i.e. the eigenvalues of the true between-group covariance matrix, see Simulation result section). Through iterative improvements of the feature space model, this approach allows the researcher, in principle, to determine the most likely distribution of information across feature dimensions.

### Random subspace approach

A second way to improve the sensitivity of the method is to combine the output of multiple classifiers, each trained on a slightly different random subset of the voxel in an area of interest. Whereas each individual classifier is noisier than the one trained on all voxels, their average performance is often more stable and reliable than the single classifier (Rokach, 2010).

Typically, such a random subspace approach would be performed on a region of interest (ROI), for which we seek to determine the dimensionality. To compare the random subspace approach with one that simply uses all the data, we generated an ROI of 400 voxels with a 1-, 2- or 3-dimensional feature space, each with matched accuracy for the full classifier. We then determined the ability of the set of classifiers that were trained on all 400 voxels to determine the dimensionality of the underlying data. In 57.2% of the simulations the best performing classifier matched the dimensionality of the underlying representation.

For the random subspace approach, we repeatedly drew subsets of 80 voxels without replacement from the 400 voxels, and averaged the classification accuracy for the 1- to 3-dimensional classifier over 2000 draws. While the classifier based on the smaller subspace of voxels had lower accuracy on average (61.3% vs. 70.6% for the full classifier), the standard deviation of the average random subspace accuracy was 20–30% lower than for the classifiers that use all the data. Correspondingly, the percentage for simulation for which we could correctly identify the dimensionality of the representation increased to 61.2%. Thus, taking samples of smaller size from the larger ROI and repeatedly running the classification analysis can be beneficial for a more reliable assessment of the accuracy curve.

### Voxel selection

The selection of voxels for decoding analysis requires caution, as the validity of the analysis hinges critically upon the fact that any criterion by which voxels are selected is independent of the data that is to be decoded (the test data). This is also the case when we try to estimate the dimensionality of representations using the proposed method. Fig. 4 shows a simulation of the influence of voxel selection onto the accuracy curves. Here we simulated a region with 534 voxels. For each simulation run, we selected the 15% most informative voxels—i.e. the voxels with the highest statistical value for an F-test across categories. These voxels were then submitted to the dimensionality analysis. As can be seen from Fig. 4, this approach leads us to believe that the representation was 3-dimensional, no matter whether the true representation was 1-, 2-, or 3-dimensional. This effect arises because there will be – among the selected voxels – many for which the high F-value results from noise. Because these voxels will distinguish between the conditions along randomly selected – and hence different – dimensions, this will lead to a well-spaced arrangement of the conditions in feature space.

If it is necessary to select voxels from weakly informative regions, then this selection needs to be done within the cross-validation procedure. The dashed line in Fig. 4 shows an additional simulation, in which we selected the best 80 out of 534 voxels on each cross-validation run separately. Importantly, the F-values, upon which the voxels were selected, were calculated only on the training set. In this case, the dimensionality analysis remains valid, with the highest accuracy observed when the dimensionality of the classifier matched the dimensionality of the data. To summarize, voxel selection can seriously bias the representational analysis and should in general be avoided. If voxel-selection becomes necessary because the statistical power is too low otherwise, it needs to be performed in a cross-validated fashion.

### Representation of force levels (Experiment 1)

We then applied our method to two real data sets; chosen because we had a strong a-priori hypothesis that the dimensionality of the underlying feature space should differ between the two data sets. For the first example, we chose a neural representation for which the feature space should be relatively low-dimensional. To obtain this data set, we recorded the activity patterns in the primary motor cortex when participants pressed with all fingers of the hand at 4 different force levels (see Representations of force levels (Experiment 1) section). Many neurons in primary motor cortex increase firing rates with increasing force levels (Evarts et al., 1983). We therefore expected that the BOLD activity should also increase monotonically.

The exact dimensionality of force representations is unknown, however one may expect that the representation for the above task is one-dimensional. This would be the case if the motor system achieves different force levels by scaling of activity of the same group of neurons. Importantly, this prediction does not rest on the assumption that the BOLD signal in M1 increases linearly with the force level (Dai et al., 2001). Indeed, some authors have argued BOLD signal and force have a logarithmic relationship (Dettmers et al., 1995, 1996a,b). A one-dimensional representation only implies that all voxels scale their activity linearly with the same latent feature, which in turn may be non-linear functions of force. Furthermore, every voxel can have a different intercept and slope with the hidden feature (and therefore even may show decreasing activity with force); it is only necessary that every voxel shows the same type of non-linear relationship with force.

Alternatively, if higher force levels lead to increased recruitment of new neuronal elements, the motor cortical activity patterns associated with different force levels may occupy a higher dimensional space. Thickbroom et al. (1998) reported that static force production led to an increase in the recruitment of voxels along the central sulcus, whereas the average activity level of the activated voxels only increased slightly. Simulating a representation in which the different force levels differ in the number of activated voxels, but not in the strength of the activation, results in an accuracy curve that lies – for four different force levels – between the 3-dimensionally randomly space and 3-dimensionally evenly spaced representations (Fig. 3).

**Fig. 5:**
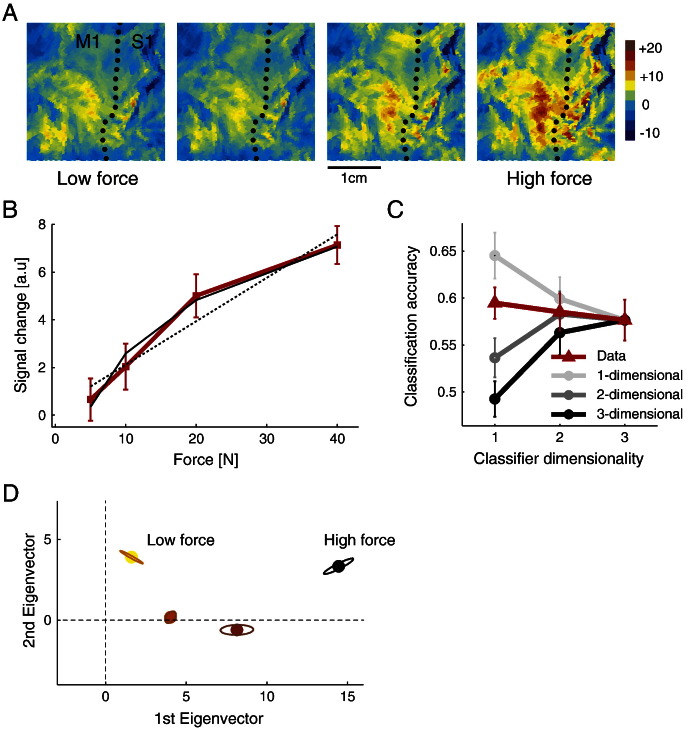
Variation of activity patterns in primary motor cortex with different force levels (Experiment 1). (A) BOLD-activity patterns in the hand area of the primary motor cortex in a flat representation. Activity patterns for a single individual are shown. The dotted line indicates the fundus of the central sulcus while the bend in sulcus indicates the location of hand knob. Color maps indicate t-values of BOLD activation against rest, for low (left panel), medium (middle two panels) and high (right panel) force levels. (B) Bold-activity (red) averaged within each M1 ROI and averaged over participants, with the error bars indicating between-subject standard error. Activity increases nonlinearly with the level of exerted force. The linear (dashed line) and logarithmic (solid line) fits are shown. (C) Classification accuracy for classifiers of different dimensionality. Average results for 5 subjects are shown (red line). Predicted accuracies based on simulations with randomly spaced 1- to 3-dimensional representations are shown in gray. The simulations are matched to the data to have the same accuracy of the full-dimensional classifier. Error bars indicate between-subject standard error. (D) Average arrangement of activity patterns in an inferred feature space, defined by the 1st and 2nd most discriminating eigenvector. Ellipses indicate standard deviation after alignment of individual patterns using the Procrustes analysis.

Fig. 5A shows an example of the contralateral hand area of an individual participant. The hand-knob (Yousry et al., 1997) is clearly indicated by the bend in the central sulcus. For each participant, the main motor-cortical activity during the hand press was located close to this landmark. As can be seen, the signal intensity of the pattern increases with the amount of force, without the pattern changing its overall shape. The group-averaged data confirms the monotonic increase of the overall activity with force level (Fig. 5B). For the lower force levels this increase was close to linear, whereas it saturated for higher force levels. Thus, we found a force-BOLD relationship that lay between a linear (Dai et al., 2001) and logarithmic (Dettmers et al., 1996a) relationship, with the logarithmic model fitting the data better than a linear model.

Independent of the exact profile of the non-linearity, our method tries to establish whether the *activity pattern* scales linearly with this (possibly nonlinear) latent feature, or whether the pattern changes qualitatively with increasing force. To generate the accuracy curves, we defined an M1 ROI based on the probabilistic cytoarchitectonic maps (see Basic data analysis section). Within this ROI we employed a random subspace approach, each time sampling groups of 80 voxels. The average accuracy curve across these random samples (2000 times per subject, red line, Fig. 5C) was highest for the 1-dimensional classifier, suggesting indeed that the activity patterns are (up to an intercept) scaled versions for each other.

We then compared the empirical accuracy curves to simulated accuracy curves coming from a 1-, 2- or 3-dimensional representation, generated such that the classification accuracy for the full classifier matched the real data (Fig. 5C, gray lines). This comparison showed that the accuracy for the 1-dimensional classifier was not as good as expected from a truly 1-dimensional representation, (t(4) = − 5.98, p = 0.004), but is better than would be expected from a randomly-space 2-dimensional representation, (t(4) = 3.08, p = 0.037). Most certainly, however, the empirical accuracy curves were not consistent with a pure recruitment hypothesis (Thickbroom et al., 1998). Such a representation should have led to an accuracy curve with much poorer performance for the one-dimensional classifier.

Thus we must conclude that the dimensionality of the representation lies between a 1- and a 2-dimensional representation, with more variability explained by the first feature dimension than expected from a random placement of force levels in a 2-dimensional feature space. The deviation from a purely one-dimensional representation reflects a slight qualitative change of the underlying spatial activity pattern with force levels. Consistent with this conclusion, we found that for 3 out of 5 participants the 2-dimensional classifier performed better than the 1-dimensional classifier.

To investigate the representation in more detail, we projected the mean activity patterns for each condition onto the first two dimensions of the representation (see Methods). This projection (Fig. 5D) shows a highly consistent arrangement of the activity patterns across participants. The first dimension simply orders the different force levels, consistent with the overall increase in activity. The second dimension arranges the points in a quadratic pattern, whereas the third dimension (not shown here) arranges them in a cubic pattern (zig-zag) pattern. Thus, these higher dimensions consistently captured the smaller non-linearities of the scaling of the activity patterns in the nonlinear feature space.

In sum, our results indicate that the activity patterns in primary motor cortex increase – to a first approximation – linearly with a latent feature that, in turn, scales (at least for higher force levels) nonlinearly with force. Our results are clearly not consistent with a recruitment hypothesis—i.e. the idea that higher force levels activate more voxels, but do no change the activity level of the activated voxels.

### Representation of individuated finger presses (Experiment 2)

As an alternate example, we investigated the activity patterns elicited by individual presses of the 5 fingers of the left and right hands (see the Representation of individual finger presses (Experiment 2) section, and also Diedrichsen et al., 2012). From a behavioral standpoint we know that the participants are able to produce force with each individual finger, without pressing down with the other fingers. For isometric presses of the instructed finger of > 2.3 N, the co-activation of the other fingers was on average only 0.08 N. We have the strong a-priori hypothesis that this behavior is controlled from the hand area of primary motor cortex, and that we therefore should find a full (i.e. 4-dimensional) representation of these five behaviors.

**Fig. 6:**
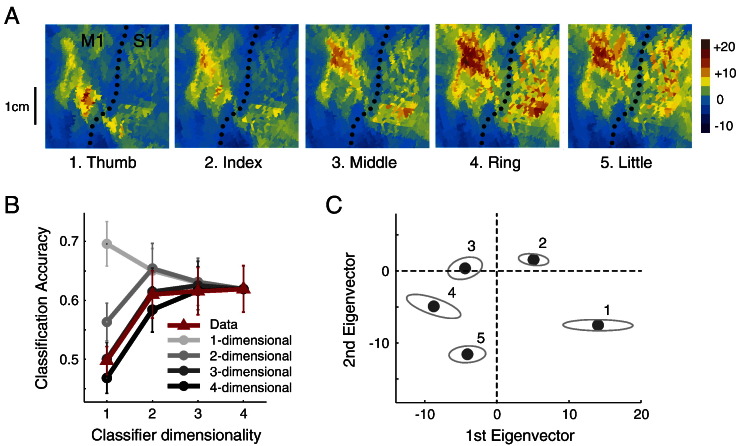
Variation of activity patterns in primary motor cortex for different finger movements (Experiment 2). (A) BOLD-activity patterns in the hand area of the primary motor cortex in a flat representation. Activity patterns for a single individual and hemisphere are shown. The dotted line indicates the fundus of the central sulcus, with the bend in the sulcus indicating the location of the hand knob. Color map indicate t-values of BOLD activation against rest, for digit 1–5 (left to right). (B) Classification accuracy for classifiers of different dimensionality. Average results from 12 hemispheres of 6 participants are shown in red. Predicted accuracies based on simulations with randomly spaced 1- to 4-dimensional representations are shown in gray, and matched for the accuracy of the full-dimensional classifier. Error bars indicate between-subject standard error. (C) Average arrangement of activity patterns in an inferred feature space, defined by the 1st and 2nd most discriminating eigenvector. Ellipses indicate standard deviation after affine alignment of individual patterns.

Fig. 6A shows the activity patterns associated with each finger in an individual participant. As expected from neurophysiological results (Schieber, 2002; Schieber and Hibbard, 1993), we found highly overlapping patches of activity for the 5 fingers. Secondly, we observed that the activity was highest for the ring finger, a finding that held true for the average group data. However, we can also see that the patterns did not only differ in intensity, but also in their spatial shape: A thumb press led to more ventro-lateral activity, in contrast to more dorso-medial activation for the little finger (Indovina and Sanes, 2001; Wiestler et al., 2011).

The accuracy curve (Fig. 6B) showed that the highest cross-validated accuracy was observed for the 4-dimensional classifier. This was true in the group mean, and also for 9 out of the 12 studied hemispheres. These results show that, as expected, the motor cortex has a full representation of individual finger movements, i.e. that each finger press is associated with a unique activation pattern. We then again compared the obtained classification accuracy against the simulated counterparts, based on the assumption that each finger is represented by a random, independent activation pattern in M1. While the empirical accuracy for the one-dimensional classifier was clearly lower than would be expected for a one- or two-dimensional representation, the accuracy was also slightly higher than would be expected from a randomly-spaced 4 dimensional representation, (t(11) = 2.510 p = 0.029). Indeed, for the accuracy of the one-dimensional classifiers the simulation based on a 3-dimensional representation provided the best match.

This finding indicates that the first dimension captures more between-finger variance than would have been expected from a random spacing of finger activation patterns in feature space. Indeed, even in the raw patterns one can observe that the activity patterns for middle, ring and little fingers were quite similar to each other (Fig. 6A). The systematic arrangement can be seen clearer when projecting the average activity patterns onto the first 2 eigenvectors of the extracted feature space (Fig. 6C). While the thumb pattern is quite distant to the other activation patterns, the ring, middle and little fingers are located close together in feature space. This clustering could be introduced either by similarity of the required muscular activation patterns for individual finger presses, or could be related to the natural statistics of movement—i.e. the fact that these fingers usually move together in the main grasping synergy (Ingram et al., 2008).

Overall, however, the dimensionality analysis reveals a full representation of individuated finger movements. This stands in contrast to the clearly reduced dimensionality in the case of different force levels.

## Discussion

The concept of latent feature spaces is a powerful tool to describe neural population activity. These representational spaces are most often defined in such a way that the observed activity of each element of a population code (i.e. individual neurons for single-cell recording, or cortical assemblies in the case of LFPs or fMRI) can be described by a linear combination pattern components, each related to an underlying feature (Naselaris et al., 2011). Feature spaces have been introduced to investigate the temporal dynamics of population activity during action preparation and execution (Churchland et al., 2007, 2012), or to investigate the semantic structure of visual representation in temporal cortices (Kriegeskorte et al., 2008b; Naselaris et al., 2009).

Feature spaces can be abstract to a certain degree; their dimensions do not necessarily represent known physical quantities. This does not imply, however, that we cannot study the structure of these spaces. A description on the level of feature spaces can provide a concise characterization of the population activity and its dynamics. Here we concentrate on one fundamental characteristic: the dimensionality of the feature space. The dimensionality refers to the number of pattern components needed to describe the population activity over a set of experimental conditions, i.e. the number of non-zero eigenvalues of the *true* between-class covariance matrix. The eigenvalues of the *estimated* between-class covariance matrix, however, are unfortunately not directly informative: Due to noise, there will always be *K-1* non-zero eigenvalues for *K* experimental conditions. To address this problem we suggest here to use a cross-validated classification method. By comparing the classification accuracy of linear classifiers each based on different numbers of dimensions, the method estimates how many eigenvectors are necessary to explain the *meaningful* variability in the data (i.e. variability that distinguishes the conditions of interest).

A very related multivariate analysis technique is the evaluation of the (dis-) similarity structure of the representations, as proposed by Kriegeskorte et al. (2008a,b). As the method proposed here, representational similarity analysis enables us to evaluate the structure of the feature space without needing to pre-specify the meaning of the different dimensions. The use of multi-dimensional scaling (MDS) also allows the visualization of a low-dimensional projection of the feature space, very much as done here (see Eq. (10), Figs. 5D, 6C). However, the proposed method provides an important addition. It determines how many dimensions are needed to faithfully represent the empirically observed neural activation patterns for a given task over a cortical region, and therefore allows us to decide between different *classes* of representational hypotheses.

It should also be clear from Fig. 2 that dimensionality is not a categorical concept; rather, it is possible to see a gradual transition from cases where most of the information is carried by the first few dimensions (Fig. 2B) to cases where the information is distributed across many dimensions (Fig. 2C). The normalized eigenvalues of the true between-class covariance matrix reflect the distribution of conditions in feature space. If all dimensions have the same eigenvalue, then the different experimental conditions are separated evenly among all dimensions of feature space (Fig. 2C), implying optimal encoding of the underlying variable in the population activity. Although the eigenvalues of the true between-class covariance matrix cannot be directly estimated, we can recover some of the information by comparing the full accuracy curves of the data (from classifiers of different dimensionality) to those obtained by simulations with varying arrangements of the conditions in feature space (see Comparing full accuracy curves section).

We then applied the methods to two examples of motor cortical activation patterns. First, we found that activity patterns of reflecting different force levels were relatively well classified by a one-dimensional classifier. Thus, to a first approximation, the activity patterns differ only in their relative signal intensity. This finding clearly contradicts the hypothesis that increasing force levels are mostly characterized by an increasing number of activated voxels (Thickbroom et al., 1998). This hypothesis would have predicted very poor accuracy for the one-dimensional classifier. While the activity patterns increased nearly linearly with the latent feature, the latent feature itself was not a linear function of force. Rather, we found that the relationship between force and latent feature was linear for the lower force levels, but saturated for the highest force level.

Secondly, we found that the hand area of motor cortex has a full-dimensional representation of individuated finger movements. A 4-dimensional classifier performed better than lower dimensional classifiers in distinguishing between the activity patterns for each of the 5 fingers. This indicates that each finger press is associated with its own unique pattern of activity. This was to be expected, given that the primary motor cortex is responsible for producing individuated finger movements (Porter and Lemon, 1993). Our results, however, also indicate that the fingers are not represented in terms of exclusive and highly separable patches. This would have resulted in an even spacing of the fingers in feature space, and hence to a poor performance of the lower-dimensional classifiers. Rather, our data suggests that a considerable amount of between-finger information is contained in the first two dimensions of the feature space. This may result from the fact that the movements of the ring, middle and little fingers are represented very closely together in feature space, as these three fingers most often act together during natural movements (Ingram et al., 2008) and show significant co-activation at higher force levels (Zatsiorsky et al., 1998).

These findings may not be surprising by themselves; but they show that the proposed method produces clearly interpretable results when applied to real neuroimaging data. Given this validation, we believe that the method can now be applied in experiments with more complex actions or stimuli. For example, we may wish to know which regions represent more complex variables, such as the ordering of finger presses in a sequential task, the emotional content of facial expressions, or the identity of visual objects. For such stimuli, one can often find a number of regions that exhibit above-chance classification accuracy. One problem with the interpretation of such findings is that one cannot be sure whether the region represents the experimental conditions by associating each condition with a unique pattern (a full-dimensional representation), or whether it simply scales the activity with a single latent variable. While careful experimental design should ensure that the experimental conditions do not differ dramatically on possibly confounding dimensions, such as in the brightness of the stimulus or the difficulty of the task, perfect matching of the conditions can never be achieved. For example, different actions always differ slightly in the required effort, and while one could try to equate these differences, this can only be achieved by introducing differences in other variables. Ultimately, even with the best design, one can never rule out convincingly that the classification was not performed on a single latent (possible unobservable) variable. The new method now offers the possibility to distinguish in general between full-dimensional representations, and those in which activity simply scales with a few latent variables. While it is also theoretically possible to test for specific dependencies by regressing out the confounding variable of the data prior to classification, our proposed technique is more versatile, as it can test whole classes of alternative hypotheses.

Apart from evaluating the alternative hypothesis that above-chance classification performance arises from a low-dimensional (and hence artifactual) representation, we believe that the method will also be useful to determine the structure of more complex feature spaces. For example, we do not know how the primary motor cortex represents (and controls) complex hand movements. While it has been hypothesized that hand movements are assembled by a finite set of synergies (Gentner and Classen, 2006), the number, shape, and neural implementation of these hypothetical building blocks remain unknown. To study such representation, it will be necessary to acquire neural data from a broad range of hand movements. The ultimate goal is to build quantitative models, which can explain and predict the neural data from such condition-rich designs (Kriegeskorte et al., 2008a). Such models however, require an educated guess about the number and types of features an area might code for (Kay et al., 2008). In this process, our method can provide the first step by determining the number of necessary dimensions and the distribution of information across these dimensions.

While we focus here on the analysis of fMRI data, similar techniques can also be applied to neural population data from other modalities, such as recordings of local field potentials or data from multi-electrode arrays. In the process of building quantitative models of neural population activity, the proposed method fills an important gap in the neuroscientific toolkit. On the one hand it goes beyond purely data-driven approaches (such as principle component analysis or other methods for dimensionality reduction), as it tests specific hypotheses about the dimensionality of the underlying feature space. On the other hand, it does not require the specification of the precise form of the underlying feature dimensions and their (possibly nonlinear) relationship to physical variables.
